# Comparative Evaluation of Different Targeted and Untargeted Analytical Approaches to Assess Greek Extra Virgin Olive Oil Quality and Authentication

**DOI:** 10.3390/molecules27041350

**Published:** 2022-02-16

**Authors:** Sofia Drakopoulou, Emmanouil Orfanakis, Ioulia Karagiannaki, Fragiskos Gaitis, Stavroula Skoulika, Andreas Papaioannou, George Boukouvalas, George Petropoulos, Vassilios Katsoudas, Renate Kontzedaki, Aggelos Philippidis, Aikaterini Zoumi, Marilena Dasenaki, Nikolaos S. Thomaidis, Michalis Velegrakis

**Affiliations:** 1 Laboratory of Analytical Chemistry, Department of Chemistry, National and Kapodistrian University of Athens, Panepistimiopolis Zografou, 15771 Athens, Greece; sofiadrakop@chem.uoa.gr (S.D.); mdasenaki@chem.uoa.gr (M.D.); 2 Institute of Electronic Structure and Laser, Foundation for Research and Technology-Hellas (IESL-FORTH), 70013 Heraklion, Greece; morfanakis@iesl.forth.gr (E.O.); ikaragian@iesl.forth.gr (I.K.); renatekontzedaki@iesl.forth.gr (R.K.); filagg@iesl.forth.gr (A.P.); azoumi@iesl.forth.gr (A.Z.); 3 Department of Materials Science and Technology, University of Crete, 70013 Heraklion, Greece; 4 Food Analytical and Research Laboratories of Athens, Directorate of Laboratories, Hellenic Food Authority (EFET) 143 42, 31 Anagenniseos Str, Nea Philadelfeia, 11526 Athens, Greece; fgaitis@efet.gr (F.G.); sskoulika@efet.gr (S.S.); apapaioannou@efet.gr (A.P.); gboukouvalas@efet.gr (G.B.); gpetropoulos@efet.gr (G.P.); vkatsoudas@efet.gr (V.K.); 5 Laboratory of Food Chemistry, Department of Chemistry, National and Kapodistrian University of Athens, Panepistimiopolis Zografou, 15771 Athens, Greece

**Keywords:** extra virgin olive oil, authenticity, variety identification, FAMEs, HRMS, metabolomics, optical spectroscopy, visible absorption, fluorescence, Raman, machine learning

## Abstract

Extra virgin olive oil (EVOO) is a key component of the Mediterranean diet, with several health benefits derived from its consumption. Moreover, due to its eminent market position, EVOO has been thoroughly studied over the last several years, aiming at its authentication, but also to reveal the chemical profile inherent to its beneficial properties. In the present work, a comparative study was conducted to assess Greek EVOOs’ quality and authentication utilizing different analytical approaches, both targeted and untargeted. 173 monovarietal EVOOs from three emblematic Greek cultivars (Koroneiki, Kolovi and Adramytiani), obtained during the harvesting years of 2018–2020, were analyzed and quantified as per their fatty acids methyl esters (FAMEs) composition via the official method (EEC) No 2568/91, as well as their bioactive content through liquid chromatography coupled to high resolution mass spectrometry (LC-HRMS) methodology. In addition to FAMEs analysis, EVOO samples were also analyzed via HRMS-untargeted metabolomics and optical spectroscopy techniques (visible absorption, fluorescence and Raman). The data retrieved from all applied techniques were analyzed with Machine Learning methods for the authentication of the EVOOs’ variety. The models’ predictive performance was calculated through test samples, while for further evaluation 30 commercially available EVOO samples were also examined in terms of variety. To the best of our knowledge, this is the first study where different techniques from the fields of standard analysis, spectrometry and optical spectroscopy are applied to the same EVOO samples, providing strong insight into EVOOs chemical profile and a comparative evaluation through the different platforms.

## 1. Introduction

Extra virgin olive oil (EVOO) has attracted particular attention due to its high nutritional value. Mainly consisting of triacylglycerols, which represent more than 98% of the oil weight, and small quantities of free fatty acids (less than 0.8% in EVOOs [1]), it is considered one of the most valuable edible fats worldwide, with several health benefits being derived by its consumption. Olive oil consumption has been associated with the prevention of cardiovascular disease, diabetes, cancer, age-related cognitive decline, and lower incidence of metabolic syndrome [2]. In fact, bioactive compounds included in the minor fraction of 2% *w*/*w* together with monounsaturated fatty acids (MUFAs), mainly referring to oleic acid, which is the most predominant type, present an increasing potential for health protection by decreasing low-density lipoprotein levels [3,4]. In this ambit, acknowledging EVOO’s high nutritional value and the health benefits derived from its consumption, European Union (EU) has proceeded to the establishment of relative regulation frameworks and health claims. Specifically, regarding fatty acids, the EU health claim of 2006 acknowledges cis-MUFAs (e.g., oleic acid) and cis- polyunsaturated fatty acids (PUFAs) (e.g., linoleic acid and alpha-linolenic acid) to the maintenance of normal blood cholesterol levels [5]. In the same aspect, the regulation (EU) 432/2012 stated that foods with concentration of alpha-linolenic acid above 0.6 g/100 g are considered as foods with high content in Ω-3 fatty acids, whereas foods with concentration of linoleic acid above 1.5 g/100 g helps maintain normal blood cholesterol levels [6]. The same regulation also highlights the health effects related to polyphenols, directly associated with European Food Safety Authority (EFSA) preceded substantiation about the benefits of EVOOs consumption [7], setting the limit of “at least 5 mg of hydroxytyrosol and its derivatives (e.g., oleuropein complex and tyrosol) per 20 g of olive oil” [6]. Different methodologies have been developed to assure that olive oil meets regulatory standards and to allow the use of health claims related to fatty acids and bioactive content. Regarding olive oil quality assurance, the methodologies applied by all control authorities are determined by the protocols established from regulatory bodies (e.g., European Commission Regulation) [1]. 

Under the perspective of authenticity assessment and quality verification, more holistic approaches are needed in order to address challenging authenticity issues, such as variety or geographical origin identification of EVOO. High resolution mass spectrometry (HRMS)-metabolomics and optical spectroscopy are two cutting-edge methodologies that make full use of EVOOs chemical profile, allowing their thorough investigation. HRMS-metabolomics has demonstrated its potential on the field of EVOOs analysis with both targeted (focusing on a specific group of compounds-metabolites, identified and quantified) and untargeted approaches (investigating the whole profile) being reported so far [8,9,10,11,12]. HRMS-workflows, often hyphenated with chromatographic techniques (e.g., liquid chromatography, LC), increase depth of coverage, with compounds of different chemical classes being detected in a single acquisition run. Hence, the methodology developed and applied by the authors [8] regarding EVOOs’ bioactive content determination, allows the detection of a large number of compounds separated according to the different chemical groups (i.e., phenols, lignans, terpenoids, flavonoids, fatty acids etc.). As a result, a holistic overview on EVOOs profile is implemented, which is crucial in metabolomics-based studies. Moreover, taking advantage of HRMS data dependent acquisition (DDA) mode, high-quality spectra are provided, thus enabling the accurate identification and structure annotation of even unknown compounds. To this purpose, the followed data treatment workflow comprises all information retrieved through LC-HRMS analysis (i.e., retention time, mass accuracy, isotopic pattern and MS2 fragmentation) aiming for tentative identification.

Optical spectroscopy has been introduced as a powerful analytical tool for EVOO authenticity determination, providing rapid and accurate results. Spectroscopic techniques such as absorption, fluorescence, Raman and FT-IR are non-invasive methods, relatively cheap, and user and environmentally friendly, as they have the advantage of the absence of reagents and solvents and require a small amount of sample. Over the last several years, the application of optical spectroscopic techniques combined with machine learning methods has been used to deal with various analytical problems of interest in the olive oil authenticity sector. Specifically, optical spectroscopic techniques were used to distinguish the EVOO samples based on their geographical origin [13]. In addition, they have been used for detection of adulteration in extra virgin olive oil [14] and for the verification of its quality parameters [15,16]. Moreover, these techniques have also been shown to be efficient tools for the identification of EVOOs based on their variety [17,18]. 

Both spectrometry and optical spectroscopy techniques derive data which contain a large amount of information. In order to eliminate noise and redundant information, machine learning analysis is considered a powerful and essential tool. Machine Learning methods, and in this case classification methods, give insight and intuition into the points of interest (i.e., identification of variety, geographical or botanical origin). Both targeted and untargeted techniques have been previously combined with machine learning methods, such as PLS-DA, SVMs, Neural Networks etc. Indicatively, various agricultural products such as honey [19,20] and wine [21,22,23,24,25,26] have been investigated, considering a wide range of issues and authentication purposes such as their quality, variety and geographical and botanical origin.

In the present collaborative study, both targeted and untargeted approaches have been established to evaluate olive oil quality and assure its authentication in terms of variety identification. Greek EVOOs have been analyzed and assessed to meet the established health claims, thus highlighting their nutritional value, while thorough study of their profile has also been performed. Regarding the latter, both HRMS and optical spectroscopy techniques were implemented to gain perspective and investigate different chemical classes. All information retrieved was utilized to its fullest, contributing to the creation of predictive models, according to the parameter of analysis (i.e., fatty acids content, metabolomics, visible absorption, Raman and fluorescence). For the construction of the predictive models, the most widely used classification methods were employed, namely the Logistic Regression, Random Forest, SVMs and KNN. For each different technique (FAMEs, HRMS, visible absorption, Raman and fluorescence), the best-performing classification model was chosen based on the cross-validation procedure. Each model was validated and evaluated for its ability to correctly discriminate between three emblematic Greek olive oil varieties (Koroneiki, Kolovi and Adramytiani), performing a comparative study through the different analytical platforms. Finally, the constructed models were further evaluated with 30 commercially available EVOO samples in order to verify their variety.

## 2. Results and Discussion

### 2.1. Fatty Acids Methyl Esters (FAMEs)

**Figure 1:**
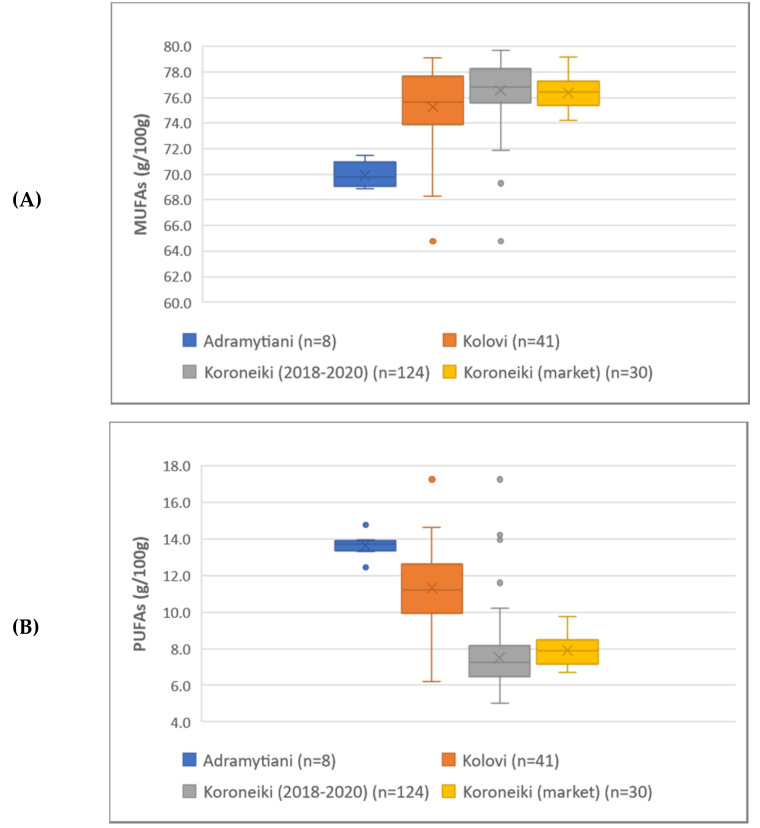
Box-and-whisker plots of EVOOs from the three different varieties regarding MUFAs (**A**) and PUFAs (**B**).

**Figure 2:**
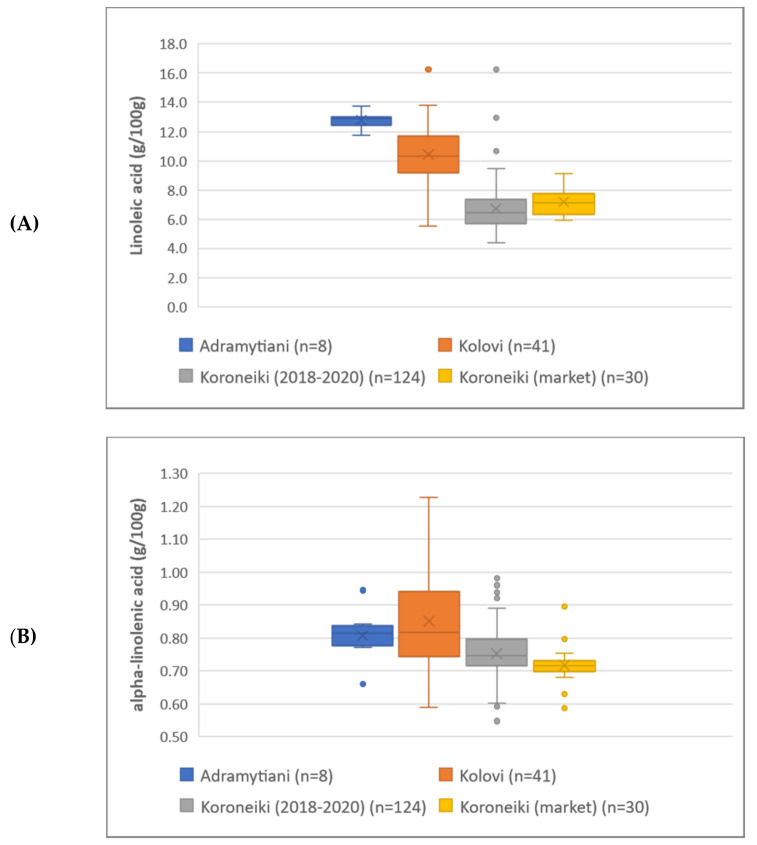
Box-and-whisker plots of EVOOs from the three different varieties regarding linoleic (**A**) and alpha-linolenic acid (**B**).

In total, 17 different fatty acids, in their methyl-esterified form, were detected and quantified in all olive oil samples. FAMEs have been characterized as MUFAs and PUFAs based on their saturation (Table S1), while linoleic and alpha-linolenic acid were also studied individually according to their health claims established. As indicated from the box-and-whisker plots of MUFAs and PUFAs (Figure 1A,B), the samples of Koroneiki variety were found to be rich in MUFAs (i.e., mostly oleic acid), while the EVOOs of Adramytiani and Kolovi variety prevailed in PUFAs content (i.e., linoleic and alpha-linolenic acid). Regarding linoleic acid and alpha-linolenic acid in particular (Figure 2A,B), the entire set of samples was found to be rich in content of both fatty acids, thus highlighting Greek EVOOs’ high quality and nutritional value. More specifically, a mean value of 9.28 g/100 g in linoleic acid was recorded, 6.2-fold higher than the limit reported in the Regulation (referring to 1.50 g/100 g), while alpha-linolenic acid mean value was found to be 0.78 g/100 g, 1.3-fold higher compared to the regulation limit of 0.60 g/100 g. Analysis of variance (ANOVA) was also performed to study potential differentiation among groups, resulting that EVOOs from the three different varieties are indeed differentiated, with the mean difference between the groups being statistically significant (Table S2). Koroneiki’s market samples are intentionally depicted separately in the chart for comparison purposes, with the mean value though not being statistically significant compared to those of Koroneiki sampling 2018–2020 (Table S3). All statistical information regarding FAMEs’ results is provided in Table S4.

The FAMEs content of EVOOs, including all 17 fatty acids, were consequently combined with machine learning methods to investigate the variety identification. SVM was selected as optimum classification method with RBF kernel and regularization parameter C = 1.5. Table 1 presents the outcome of the classification, with all 52 test samples being correctly classified based on their variety, recording the total score of 100%, both on accuracy, mean sensitivity and mean specificity.

### 2.2. HRMS-Metabolomics

#### 2.2.1. Bioactive Content

**Figure 3:**
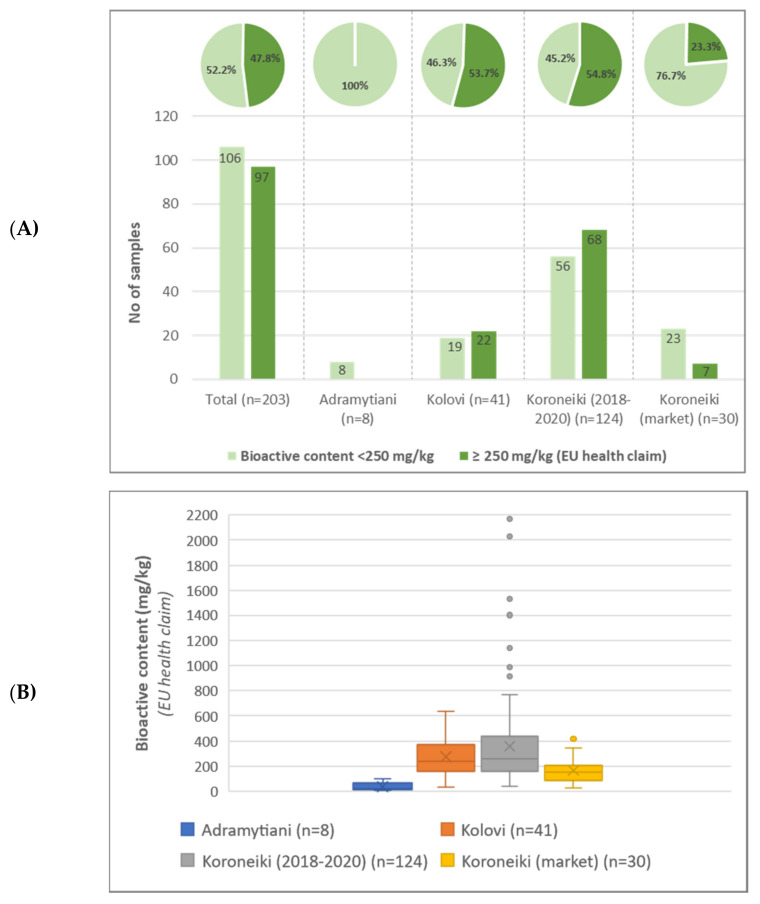
Column and pie-charts depicting the number and % percentage of EVOOs that may claim or not the EU health indication based on their bioactive content (**A**), and box-and-whisker plot of EVOOs from the three different varieties regarding bioactive content (**B**).

All samples were analyzed and evaluated regarding their bioactive content. 25 phenolic compounds available in the in-house target database were detected and further quantified (Table S5). Among them, 14 compounds were characterized as “hydroxytyrosol derivatives” and therefore related to EU health claim legislation. Figure 3 depicts EVOOs’ bioactive content evaluated for all the monovarietal samples belonging to the three different varieties as well for the market samples. Approximately, half of the samples (47.8%) encountered bioactive content higher or equal to 250 mg/kg and could consequently bear the health claim indication as described by EU 432/2012 legislation. It is also worth noticing that only 23.3% of the samples acquired from the market reached the health claim limit (Figure 3A), which may be partially attributed to the storage conditions and shelf-time of the product, parameters that highly affect EVOOs’ bioactive content [27]. ANOVA was also implemented among the groups, demonstrating that the three varieties are differentiated, with the mean difference between the groups being statistically significant (Table S6). Results from bioactive content analysis, along with the statistical parameters used in pie- and box--and-whisker plots, are summarized in Table S7.

#### 2.2.2. Untargeted Metabolomics

**Figure 4:**
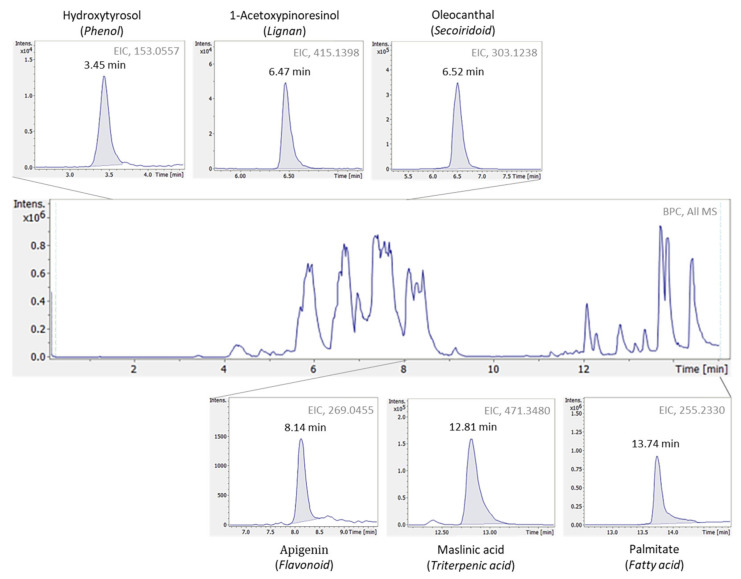
Typical base peak chromatogram (BPC) of extra virgin olive oil and extracted ion chromatograms (EICs) of compounds from different chemical classes, detected with the current untargeted HRMS methodology.

Taking a step forward, an untargeted HRMS approach was implemented aiming at EVOOs’ holistic profile evaluation. In contrast with the targeted approach which focused particularly in one chemical class (i.e., phenols and derivatives), on the applied untargeted methodology, compounds of different chemical classes were studied, increasing depth-of-coverage and enabling the study of EVOOs total profile. Figure 4 illustrates a typical base peak chromatogram (BPC), as well as extracted ion chromatograms (EICs) of compounds classified into different chemical groups, acquired with the current HRMS methodology applied in EVOOs.

All information retrieved through untargeted HRMS metabolomics was combined with machine learning methods for the identification of the three examined olive oil varieties. More specifically, model-based feature selection was performed using Random Forest with 100 trees, minimum split size = 2 and minimum leaf size = 9, while classification was carried out using Logistic Regression with l2 penalty and regularization parameter C = 0.5. As observed in the confusion matrix of Table 2, all 52 test samples were correctly classified leading to 100% accuracy, mean sensitivity and mean specificity.

#### 2.2.3. Variety Markers’ Identification

During the feature selection procedure 92 out of the total 648 exact mass retention time (EMRT) pairs were selected. The HRMS-based methodology applied in the study enabled the identification of features of interest, providing high-quality MS and MS2 spectra, inherent to accurate formula and structure assignment, respectively. Identification was performed via in-house target and suspect databases available [10]. The identification criteria have been previously described in “Data treatment” section. For unknown compounds though (i.e., features not included in the databases), a stepwise identification approach was implemented utilizing all information available from HRMS analysis. More specifically, formula annotation was performed using Bruker’s “SmartFormula Manually” tool, embedded in DataAnalysis software, which estimates formulas based on accurate mass and isotopic pattern. Only compounds consisting of C (n ≤ 50), H (n ≤ 100), O (n ≤ 20) and N (n ≤ 10) atoms, which is mainly the composition of bioactive compounds, were considered for the molecular formula assignment. Proposed formulas were sorted according to their SmartFormula Manually Scores, with the most prevailed ones scoring 100%, while the rest follow in a descending order.

**Figure 5:**
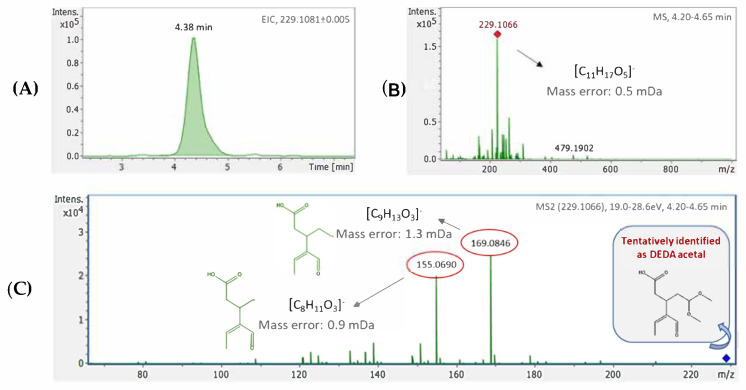
Identification for EMRT 229.1081_4.38 (DEDA acetal). EIC (**A**), MS spectrum and probable elemental composition (**B**), as well as MS2 spectrum depicting compound’s fragments along with their structure assignment (**C**).

Subsequently, a thorough search in online databases (e.g., MassBank, http://www.massbank.jp/?lang=en, accessed on 15 October 2021; ChEBI, https://www.ebi.ac.uk/chebi/, accessed on 15 October 2021) and FoodB, http://foodb.ca/, accessed on 15 October 2021) was performed to assign formula to a probable structure. Only compounds found in olive oil and products (consistent to OliveNet™ [28], https://mccordresearch.com.au/, accessed on 15 October 2021) were considered eminent candidates and further examined. MS2 fragmentation pattern was compared to those of the databases, while in cases of no experimental data available, in silico fragmentation was also assessed through MetFrag [29]. Figure 5 illustrates the identification performed in the case of DEDA acetal, while Table S8 includes the compounds tentatively identified.

### 2.3. Optical Spectroscopic Methods

#### 2.3.1. Absorption Spectroscopy

**Figure 6:**
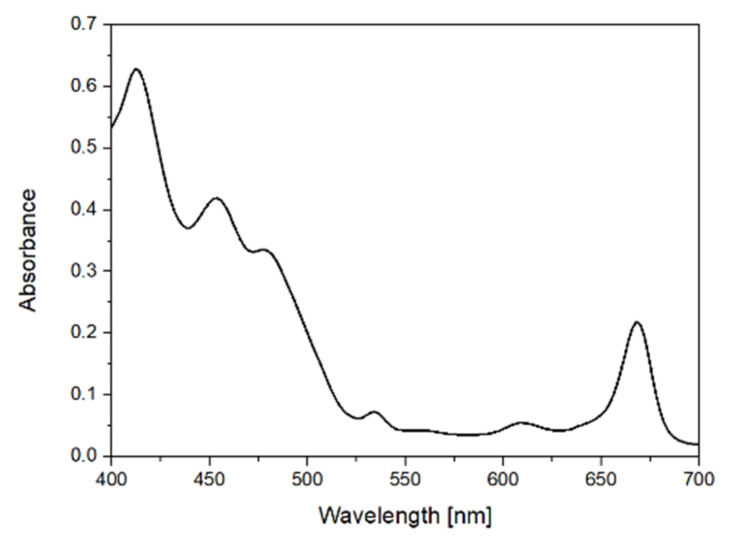
Absorption spectrum of extra virgin olive oil (EVOO) in the region 400–700 nm.

The EVOO samples from the three different varieties were analyzed by absorption spectroscopy in the visible region (400–700 nm). Figure 6 displays a typical absorption spectrum of EVOO in this region. The spectrum of EVOO in the visible region contains information mainly for pigments, such us carotenoids and chlorophyll, and the bands that appear are due to the electronic excitations’ transitions of those compounds. Specifically, the observed peaks at 454 and 480 nm are assigned to carotenoids contained in the olive oil samples. The peaks at 416, 535, 610 and 670 nm correspond to the presence of chlorophyll in the samples [13,30].

The evaluation of the absorption spectroscopic data was performed via model-based feature selection using Random Forest with 100 trees, minimum split size = 9 and minimum leaf size = 3 and via classification using SVM with linear kernel and regularization parameter C = 1.5. According to Table 3, in which the confusion matrix of the test samples is presented, 5 out of 37 samples of Koroneiki variety were misclassified. The samples from Adramytiani and Kolovi varieties were shown a 100% correct classification. In total, 5 out of the 52 test samples were misclassified. The obtained results depict a high rate of correct classification as the accuracy of this classification was 0.9 and the balanced accuracy was 0.95. During the feature selection procedure 47 features out of the total 148 were selected. The evaluation of feature selection that was applied to the absorption spectroscopic data for the presented classification indicated selected features at 400–524 nm and 580–694 nm from the spectral region 400–700 nm. As mentioned above, the selected features are related to the characteristic absorption bands of several pigments such us carotenoids (beta-carotene and lutein) and chlorophyll (a, b chlorophyll and a, b pheophytin).

#### 2.3.2. Raman Spectroscopy

**Figure 7:**
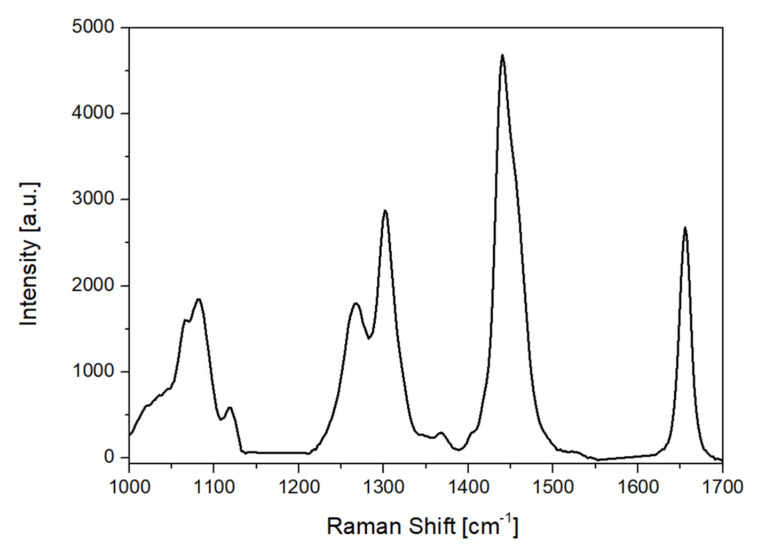
Raman spectrum of extra virgin olive oil (EVOO) from 1000 to 1700 cm^−1^ after background subtraction.

The EVOO samples from the three different varieties were also analyzed using Raman spectroscopy. Figure 7 depicts a characteristic Raman spectrum of olive oil in the range 1000–1700 cm^−1^ after the background subtraction, using the Zhangfit technique [31] Table 4 shows the characteristic Raman bands and their corresponding assignments to vibrational modes of the functional groups [13,14,32]. Those bands correspond to the fatty acids contained in the sample, with the peaks located at 1265 and 1657 cm^−1^ corresponding to the unsaturated fatty acids. The regions that were taken into account were 1000–1140 cm^−1^, 1212–1552 cm^−1^ and 1620–1700 cm^−1^ (Figure 7). The rest of the 1000–1700 cm^−1^ spectral region was excluded since it does not contain information about the varieties.

The predictive performance results of the Raman spectroscopic data are presented in this section. Based on the selected model, model-based feature selection was performed using Random Forest with 100 trees, minimum split size = 2 and minimum leaf size = 10, and classification using SVM with linear kernel and regularization parameter C = 1. Table 5 shows the confusion matrix of the test samples. As observed in Table 5, 49 out of the total 52 test samples were correctly classified leading to an accuracy score of 94%, while the mean sensitivity is equal to 85% and mean specificity is equal to 97%. The lower value regarding the mean sensitivity is related to the misclassification of one sample from Adramytiani variety, which had high influence on the balanced accuracy score due to the small sample size of this specific class. During the Feature Selection procedure, 42 from the total 234 features were selected. The selected features were mainly associated with the spectral regions at 1040–1087 cm^−1^, 1222–1275 cm^−1^ and band at 1655 cm^−1^. As seen from the Raman band assignment in Table 4, the spectral regions which mainly contributed to EVOOs variety identification are due to stretching vibrations of unsaturated fatty acids. These results are in good agreement with the FAMEs results (Section 2.1), which reveal that MUFA and PUFA mean values are significantly different for the three examined varieties.

#### 2.3.3. Fluorescence Spectroscopy

**Figure 8:**
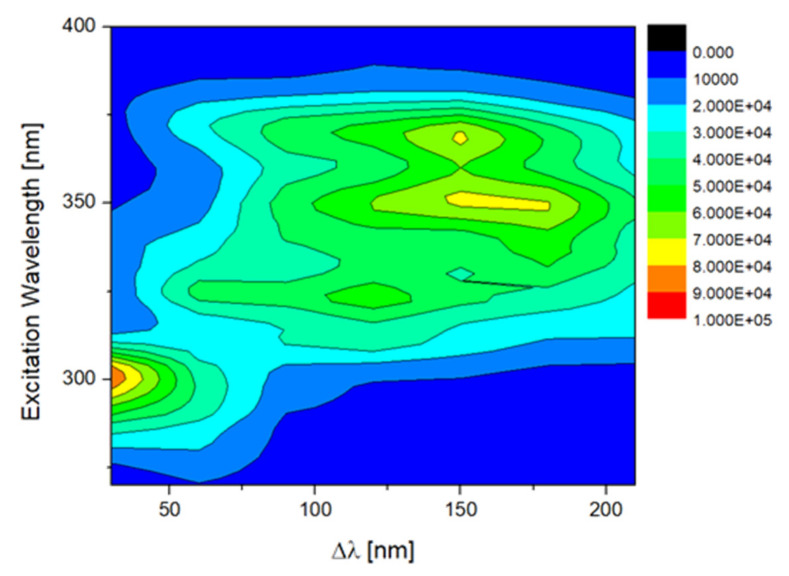
Contour map constructed by synchronous fluorescence spectra of EVOO samples. In this map, x-axis depicts the different Δλ values, y-axis depicts the excitation wavelength and the color scale represent the fluorescence intensity with blue and red corresponding to the weakest and stronger intensities, respectively.

The synchronous fluorescence spectra (SFS) of the EVOO samples for different Δλ values were measured (Section 3.3). Each SFS contained 66 data points of fluorescence intensity. These data were then used to construct a three-dimensional matrix with seven columns and 66 rows for each sample. Figure 8 exhibits a typical contour map of an EVOO sample constructed from this matrix. The contour map contains numerous emission bands and can be divided manly into two regions. The first region presents a short-wavelength characteristic band and is associated with the presence of tocopherols and its derivatives. In general, the excitation range of 270–320 nm and emission at 290–400 nm is associated with tocopherols, tocotrienols and several phenolic compounds. The phenolic compounds contribution in this region is mainly due to tyrosol and hydroxytyrosol which originated from phenolic glycosides contained in the EVOO. The second region with an excitation range of 290–360 nm and an intermediate emission range at 350–480 nm is attributed to the oxidation products [15]. Moreover, as reported in the literature, the excitation range from 300 to 400 nm with emission at 450–580 nm is due to the excitation of vitamin E and carotenoids [18]. In the spectral region that is selected to monitor the fluorescence of EVOO the intense emission of pigments such as chlorophyll is absent. Chlorophyll b, along with its derivatives (pheophytin a and b), is excited at around 350–420 nm and emits light at 650–700 nm. Due to the several fluorophores contained in the EVOO, fluorescence spectroscopy represents the fingerprint of the EVOO that reflects its chemical composition.

For the evaluation of the fluorescence spectroscopic data, each sample was unfolded into a single row, where the different scans were placed successively resulting in 7 × 66 = 462 data points (features), as reported in previous works [33]. In this way the fluorescence spectroscopic data were handled like the other spectrometric and spectroscopic data of this study. In order to analyze the unfolded fluorescence spectroscopic data, model-based feature selection was performed using Random Forest, with 100 trees, minimum split size = 2 and minimum leaf size = 2, and classification using KNN, with number of neighbors k = 3. Table 6 presents the confusion matrix, where 51 out of 52 samples were correctly classified. The calculated classification metrics for the present predictive model are 98% for accuracy, 89% for mean sensitivity and 99% for mean specificity. For the specific analysis, during the feature selection procedure 55 out of the 462 total features were selected. The selected features belong to a wide range of the different synchronous fluorescence spectra. Most of the selected features were from the synchronous fluorescence spectra with wavelength intervals (Δλ) of 30, 60, 90 and 120 nm. From the SFS spectra the compounds which emit light is associated with tocopherols and phenolic molecules, with wavelength intervals Δλ 30 and 60 nm. Respectively, with Δλ of 90 and 120 nm, the compounds of EVOO which fluorescence are related to oxidation products, vitamin E and carotenoids. 

### 2.4. Comparative Results

In this section the predictive performance efficiency of all techniques used in this study are gathered and compared. All techniques achieved significantly better results compared to the baseline, which was a model that always predicted the most frequent class, in this case Koroneiki. Such baseline achieved 71% accuracy and 33% balanced accuracy. The models based on FAMEs and HRMS analysis managed to correctly classify all the test samples, while the results of the spectroscopic techniques are also present high values of accuracy to the correct classification of EVOOs based on their variety. The comparative results from the five different techniques that used are presented in Table 7. The sensitivity and specificity for each variety are presented in Table S9.

### 2.5. Classification of Market Samples

After the model construction and evaluation on test data, it was furtherly tested for its ability to correctly identify the variety of market samples. Specifically, 30 market samples of Koroneiki variety were collected and introduced to the models. The number of correctly predicted market samples for each technique is presented in Table 8.

The models constructed with FAMEs, HRMS, Raman and fluorescence analysis data correctly predicted all the market samples available in the study, while the model constructed with the visible absorption analysis data misclassified 3 market samples. Overall, for the different approaches, the high predictive performance results, both in the test data and in the market samples, indicate that these techniques could give an intuition for the authentication of unlabeled EVOO samples and for the verification of labelled EVOO samples. 

## 3. Materials and Methods

### 3.1. Chemicals and Reagents

In fatty acids analysis, compounds were identified by the use of two commercial standards (i.e., FAME Mix C24-C22 and FAME Mix C4-C24) purchased from Supelco (Sigma, Saint Louis, MO, USA), while analytical grade methanol, heptane and potassium hydroxide were purchased from Carlo Erba Reagents. 

All standards and reagents used in HRMS analysis were of high-purity grade (>95%): p-coumaric acid, eriodictyol, pinoresinol and syringaldehyde were acquired from Sigma-Aldrich (Stenheim, Germany). Hydroxytyrosol and luteolin were purchased from Santa Cruz-Biotechnology (Santa Cruz, CA, USA), while apigenin, naringenin, tyrosol and vanillin were purchased from Alfa Aesar (Karlsruche, Germany). Ligstroside aglycone, oleacein, oleocanthal, oleocanthalic acid, oleomissional and oleuropein aglycone were obtained from Prof. P. Magiatis laboratory (Laboratory of Pharmacognosy and Natural Products Chemistry, Faculty of Pharmacy, University of Athens, Athens, Greece). The standards had been previously isolated from olive oil extracts and their structure and purity grade were evaluated by NMR analysis [34]. Methanol (MeOH) (LC-MS grade) was purchased from Merck (Darmstadt, Germany), whereas 2-propanol (LC-MS grade) was purchased from Fisher Scientific (Geel, Belgium). Sodium hydroxide monohydrate for trace analysis ≥99.9995% and ammonium acetate ≥99.0% were purchased from Fluka (Buchs, Switzerland). Distilled water was provided by a Milli-Q purification apparatus (Millipore Direct-Q UV, Bedford, MA, USA). Finally, regenerated cellulose syringe filters (RC, pore size 0.2 μm, diameter 15 mm) were purchased from Phenomenex (Torrance, CA, USA). Stock standard solutions of individual compounds (1000 mg L^−1^) were prepared in MeOH and stored at −20 °C in dark glass bottles. Working mix solutions at six different concentrations levels (0.5, 1, 2, 5, 10 and 20 mg L^−1^) were prepared by gradient dilution of the stock solutions in MeOH/H_2_O 80:20, *v*/*v* and analyzed each time at the beginning of each sequence.

### 3.2. EVOOs Samples and Sample Preparation Protocols 

A total of 173 monovarietal EVOOs produced from three cultivars (Koroneiki, Kolovi and Andramytiani) were collected from the three major olive-cultivated regions in Greece, namely, Crete, Peloponnese and Lesvos in descending order, during the harvesting periods of 2018–2020. Koroneiki variety is the cultivar more widely cultivated in Greece (found in all 3 regions mentioned), representing around 60% of the total Greek olive-growing land [35]. The widely cultivated olive variety, i.e., Koroneiki, allowed the selection of a large number of samples (154-market samples included). On the other side, Kolovi and Adramytiani cultivars are mainly cultivated in North Aegean region (the majority of which in Lesvos) with Kolovi representing the majority of cultivation in this geographical area. Therefore, during EVOO sampling a significant number of 41 Kolovi samples were selected, while only 8 monovarietal Adramytiani samples available were acquired. It also needs to be clarified at this point that only monovarietal samples were included in our study in order to study the influence of variety in EVOOs classification, which consequently reduced the number of Adramytiani samples included in the study Nevertheless, the limited number of Adramytiani samples has been addressed but also reported in previous studies [8]. Samples were packaged in dark-brown glass bottles and stored at 4 °C until analysis. Furthermore, during the same period, a total of 30 branded monovarietal (Koroneiki cultivar) EVOOs were sampled from the market.

The fatty acid composition was determined according to the official method of the Commission Regulation (EEC) No 2568/91 [1]. Briefly, the fatty acid methyl esters were prepared by vigorous shaking of 0.1 g olive oil in 3 mL heptane, with 0.2 mL of 2 mg L^−1^ methanolic potassium hydroxide in screwcap vials. After 30 min, 1 μL of the upper phase of the vial was injected into the gas chromatograph system for analysis. 

To study the metabolomic profile of olive oil, a liquid-liquid extraction with MeOH/H_2_O (80:20, *v*/*v*) used as extraction solvent was implemented, previously reported in the literature [36]. Syringaldehyde was used as internal standard (IS) at 1.30 mg L^−1^ [9]. Olive oil extracts were subjected to a 2-fold dilution with MeOH/H_2_O 80:20, *v*/*v* before analysis. Procedural blank was also prepared and analyzed to detect potential contamination. At the beginning of each instrumental sequence a six-points analytical standards’ curve was analyzed (from 0.5 to 20 mg L^−1^) to check system suitability. Quality control (QC) samples were also prepared and analyzed to ensure analytical performance. The QC samples were prepared by mixing same-volume aliquots of 15 EVOOs, derived from the 3 different varieties (5 EVOOs of each variety). A QC sample was injected at the beginning of each sequence (six times for conditioning) and also at regular intervals (every 10 injections) to monitor potential instrumental drifts. The intensity of three exact mass retention time (EMRT) pairs (*m*/*z* 153.0557_3.5 min, *m*/*z* 271.0612_7.0 min, and *m*/*z* 319.1187_5.6 min, corresponding to hydroxytyrosol, naringenin and oleacein respectively) were monitored, and their ratios to IS peak intensity were calculated to evaluate system stability, recording in all cases ratios within QC limits (±2 s) (Figure S1).

For the optical measurements no sample pretreatment was necessary.

### 3.3. Instrumental Analysis 

#### 3.3.1. Fatty Acids Determination

The fatty acids methyl esters (FAMEs) profile, expressed as % *m*/*m* using the peak area, was determined using an Agilent 7890A Chromatograph (Agilent Technologies, Santa Clara, SA, USA) coupled to a flame ionization detector (FID). The separation of fatty acid methyl esters was accomplished with a TR-FAME column, 50 m × 0.22 mm, i.d. 0.25 μm film thickness (Thermo Fisher Scientific, Waltham, MA, USA). The injector was set at 250 °C and the detector at 260 °C. The oven temperature was initially retained at 160 °C for 2 min, then raised with a rate of 1 °C min^−1^ up to 165 °C and kept for 30 min, then raised again with a rate of 3 °C min^−1^ up to 200 °C and kept for 8 min, resulting to a total run time of 57 min. Helium was used as carrier gas at a flow rate of 3 mL min^−1^. Each sample was injected twice, in split mode (50:1) with an injection volume of 1 μL, using an autosampler. FAMEs analysis was performed at Hellenic Food Authority (EFET). 

#### 3.3.2. HRMS-Metabolomics

Metabolomics analysis was carried out using an ultra-high performance liquid chromatography system (UHPLC) with an HPG-3400 pump (Dionex Ultimate 3000 RSLC, Thermo Fischer Scientific, Dreieich, Germany) coupled to a quadrupole time-of-flight mass spectrometer (QTOF) (Maxis Impact, Bruker Daltonics, Bremen, Germany). Samples were analyzed in reversed phase liquid chromatography (RPLC) using an Acclaim C18 column (2.1 × 100 mm, 2.2 μm) for the chromatographic separation obtained from Thermo Fischer Scientific (Dreieich, Germany) and preceded by a guard column of the same packaging material, thermostatted at 30 °C. The mobile phases consisted of water/methanol 90/10 (solvent A) and methanol (solvent B), both acidified with 5 mM ammonium acetate. A gradient elution program was adopted starting with 1% B (flow rate of 0.2 mL min^−1^) for 1 min, increased to 39% in 2 min and then to 99.9% (flow rate of 0.4 mL min^−1^) for another 11 min. At this point, 99.9% of B is being kept constant for 2 min (flow rate of 0.48 mL min^−1^) and then initial conditions are restored within 0.1 min, for the next 3 min; then the flow rate decreases to 0.2 mL min^−1^. Injection volume was set to 5 μL. The QTOF-MS system was equipped with an electrospray ionization interface (ESI), operating in negative mode, with the following operation parameters: capillary voltage 3500 V; end plate offset 500 V; nebulizer pressure 2 bar; drying gas 8 L min^−1^ and gas temperature 200 °C. The QTOF-MS system was operated in data independent acquisition mode (broadband collision-induced dissociation, bbCID), as well as in data-dependent acquisition mode (AutoMS/MS), and recorded spectra over the range of *m*/*z* 50–1000, with a scan rate of 2 Hz. A QTOF-MS external calibration was performed daily with the manufacturer’s solution. The typical resolving power of the instrument was ranged (FWHM) between 36,000 and 40,000 at *m*/*z* 226.1593, 430.9137, and 702.8636. HRMS-metabolomics was performed at National and Kapodistrian University of Athens.

#### 3.3.3. Optical Spectroscopy

Absorption spectra were recorded with a Shimadzu UV-1900 spectrophotometer (Shimadzu Corporation, Kyoto, Japan), which covers the spectral range from 190 to 1100 nm, as reported in a previous work by the authors [13]. For the visible region in the range of 400 nm to 700 nm, a quartz cuvette with a 2 nm pathlength was used. The corresponding spectral resolution for the absorption spectroscopy was 2 nm.

The fluorescence spectra were collected with Horiba FluoroMax-3 (HORIBA Ltd., Tokyo, Japan). For the measurements, a quartz cuvette, with a 10 mm pathlength was used and placed in the front face geometry (at 35° to the incident beam—in order to avoid inner filter effects) without any prior pre-treatment. Synchronous fluorescence spectra (SFS) were recorded by performing simultaneously scanning of the excitation and emission monochromators. In this case the emission wavelength (λ_emission_) was following the excitation wavelength (λ_excitation_) at a constant distance (Δλ) so that λ_emission_= λ_excitation_ + Δλ. For each sample seven different SFS were collected with wavelength intervals (Δλ) of 30, 60, 90, 120, 150, 180 and 210 nm and the excitation wavelength ranged from 270 nm to 400 nm, in 2 nm step. The integration time was set at 0.25 s and the excitation and emission bandwidths were 1 and 2 nm, respectively.

The Raman spectra were carried out with a mobile Raman spectrometer (HE 785, Horiba Jobin Yvon, Rue de Lille, France). The light source was a semiconductor diode laser, emitting at 785 nm. The instrumentation and the collection parameters have been described in previous work [13]. Optical spectroscopy measurements were performed at IESL-FORTH. 

### 3.4. Data Treatment

Data acquired from HRMS analysis were processed using target, suspect and un-target screening workflows. DataAnalysis 5.2 and TASQ 2.1 software (Bruker Daltonics) were used to the target and suspect approaches, while data treatment in the un-targeted approach was performed using MetaboScape® 4.0 software (Bruker Daltonics). 

The target database used in the screening workflow comprised of 70 bioactive compounds found in natural products and in olive oil in particular, classified under the chemical class of phenols (i.e., flavonoids, lignans, phenolic acids, secoiridoids or simple phenols). The database included information of compounds’ molecular formulas, pseudomolecular ions ([M-H]^−^), retention time (min), MS^2^ fragments (qualifier ions) as well as their elemental formulas. Compounds’ identification was performed setting specific matching criteria: mass accuracy (mass error ≤ 2 mDa), isotopic fitting (mSigma ≤ 50), retention time (±0.2 min in relevance to standard’s analysis), MS^2^ qualifier ions, minimum peak area threshold at 800 and minimum intensity threshold at 200, as reported in previous study by the authors [8]. Quantification of the analytes was performed through an external standard calibration curve [8].

“Suspect” compounds, often occurring in olive oil, but with no reference standard available, were successfully identified based on the aforementioned criteria, described in the targeted approach. Quantification was performed according to the in-house semi-quantitation methodology, previously reported by the authors [9], which relies on compounds chemical similarity. In total 9 “suspect” compounds were identified in olive oil samples and further quantified, namely 10-hydroxy decarboxymethyl oleuropein aglycone, 10-hydroxy-10-methyl oleuropein aglycone, 10-hydroxyoleuropein aglycone, 1-acetoxypinoresinol, elenolic acid, hydroxylated form of elenolic acid, hydroxytyrosol acetate, methyl oleuropein aglycone and syringaresinol. 

Regarding the un-targeted approach, a complete data pre-processing workflow, including peak-picking, mass calibration and time alignment was performed using Time aligned Region complete eXtraction (T-ReX) algorithm, embedded in Bruker’s MetaboScape software. In particular T-ReX 3D was used, compatible with LC-HRMS data acquired, which extracted features according to 3 particular parameters (i.e., retention time, mass to charge ratio (*m*/*z*) and intensity), resulting to the creation of a peak-list. In the peak-picking step, intensity threshold was set at 1000 counts and minimum peak length of 5 spectra was selected. Mass recalibration was automatically performed for each sample, using the same calibrant solution with that of external calibration (i.e., sodium acetate) at 0.1–0.25-time range (min). Retention time and mass range were also defined and set the same with analysis conditions (i.e., retention time range: 0.25–15 min, mass range: 50–1000 *m*/*z*). Primary ion of [M-H]^−^ was selected for negative acquisition mode. Procedural blank subtraction was also performed in the software interface to subtract false positive peaks and avoid potential contamination. Only features of 3-fold higher intensity compared to the respective intensity of the blank were taken into account and included in the final peak-list.

### 3.5. Machine Learning Analysis 

For the machine learning analysis a Python’s open-source library for Machine Learning, scikit-learn was used [37].

#### 3.5.1. Methods

##### Feature Selectiontitle

Prior to data classification, feature selection was performed. Feature Selection is the process of selecting a subset of features that is relevant to the target (i.e., geographical origin, variety identification etc.). It provides simplified models as well as a more intuitive interpretation of the model results. Among different feature selection techniques provided by scikit-learn, model-based feature selection was applied. Specifically, some learning algorithms perform feature selection as part of their overall operation [38,39]. For this scope, Random Forest was used as a feature selector. For this procedure the “SelectFromModel” function of scikit-learn was used.

Feature Selection was performed on the spectroscopic data as well as the HRMS data, which due to their nature might contain redundant information. For the FAMEs data, no feature selection was performed, as the data contain targeted information.

##### Classification

The classification procedure was performed to the data of the different techniques (FAMES, HRMS, Raman, Fluorescence, Visible). Specifically, four classification methods were examined: K Nearest Neighbors (KNN) [40], Support Vector Machines (SVM) [41], Logistic Regression (LR) [42] and Random Forests (RF) [43].

KNN assumes that the samples of each class are “close” to each other. Having the train data and their corresponding labels, each new sample is classified according to the majority class of its k-nearest neighbors.

Random forest is an ensemble model that fits multiple decision trees on different sub-samples of the initial train data. For a new sample, each tree in the Random Forest outputs a class prediction. The model finally predicts the most voted class. Moreover, the Random Forest model outputs the importance of each feature. The most important features are the ones that contribute the most to the classification results. Random Forest could also be used as a feature selector prior to the classification procedure, by selecting the most important features according to a threshold.

Logistic Regression is a generalized linear model used for classification purposes. Specifically, it fits a logistic function (i.e., sigmoid function) to the training data, that takes values in [0, 1]. For each new sample, the model outputs the probability that it belongs to the positive class based on the fitted logistic function. If the probability is over 0.5 the model predicts that the sample belongs to the positive class, or else to the negative class. The multiclass case is handled according to a one-vs-rest scheme.

Support Vector Machines for binary classification tasks aim to find a hyperplane in the p-dimensional space (p features in the data) that discriminates the samples of different classes. The support vectors are defined as the closest samples to the hyperplane and specify its position and orientation. SVMs can handle data that are not linearly separable by mapping them into a high-dimensional feature space, where they become linearly separable (kernel trick) [41]. The multiclass case is handled according to a one-vs-one scheme.

Both feature selection and classification methods require some user-defined parameters (hyperparameters). For example, KNN’s hyperparameter is the number of the nearest neighbors; the hyperparameters of SVM are the type of kernel (linear, RBF, polynomial, sigmoid) and the regularization parameter C. The hyperparameter tuning was performed during the cross-validation procedure. The different values of the hyperparameters of each method are shown in Table S10.

#### 3.5.2. Methodology

The data were randomly split into train (70%) and test set (30%) in a stratified manner such that the percentage of samples for each class is preserved as observed in the original data. The train set, total number 121 samples (87 Koroneiki, 29 Kolovi, 5 Adramytiani) was used for the model construction and the test set, total number 52 samples (37 Koroneiki, 12 Kolovi, 3 Adramytiani) was used for testing purposes.

In order to determine the model with the best hyperparameter set, stratified k-fold cross validation was performed in the train set. During the stratified k-fold cross validation the train samples are randomly partitioned into k equal sized folds. Each fold contains approximately the same percentage of samples of each class as in the train set. Each model is trained in the k-1 folds and the left-out fold is used for testing. The procedure is repeated k times, where every fold is used exactly one time as the test fold. The final estimation originates from the mean performance of all repetitions. In our case k was equal to 5.

After the model with the best hyperparameters was determined from cross-validation, it was trained to the entire train set. The model’s performance was then evaluated on the test set. The results were compared to the baseline (“DummyClassifier” of scikit-learn), which is a model that always predicts the most frequent class, in our case Koroneiki. 

As metrics of evaluation the accuracy score, the sensitivity and specificity, as well as the confusion matrix were used. Accuracy is the percentage of correctly predicted samples. The sensitivity and specificity are separately measured for each class. For each class, sensitivity (True Positive Rate) is shown in the following equation:

$$\mathrm{sensitivity}\;\left(\mathrm{TPR}\right)=\frac{\mathrm{TP}}{\mathrm{TP}+\mathrm{FN}}$$

Specifically, it refers to the proportion of samples that are correctly predicted to belong to the class (True positives-TP) among all samples that actually belong to the class (True Positives + False Negatives (TP + FN)). The specificity for each class refers to the proportion of samples that are correctly predicted that they do not belong to the class (True Negative-TN) among all samples that actually do not belong to the class (True Negative + False Positive (TN + FN)). It is shown in the following equation:

$$\mathrm{specificity}\;\left(\mathrm{TNR}\right)=\frac{\mathrm{TN}}{\mathrm{TN}+\mathrm{FP}}$$

The highest value for sensitivity and specificity is 100%. The mean sensitivity (specificity) is the average sensitivity (specificity) of all classes. The confusion matrix is also presented for further intuition of the results. It is a c-by-c matrix, where c is the number of classes. Each row represents the true label and each column the predicted one. The diagonal elements represent the correct predictions, while the non-diagonal elements represent the misclassifications. The highest performance is achieved when the diagonal elements are equal to the number of samples per class and all the non-diagonal elements are zero.

#### 3.5.3. Data Pre-Processing

In general, machine learning algorithms benefit from data pre-processing. In order to evaluate the data of each different technique, the most appropriate and required pre-processing methods were applied. Specifically, autoscale (z-score) was performed in the FAMEs data.

In HRMS data, extracted features were subjected to normalization by internal standard (i.e., syringaldehyde) to reduce variation between samples that may attributed to sample procedure, instrumental conditions, or data-processing factors.

For the Raman spectroscopic data, baseline subtraction was performed to correct the baseline offset caused by sample fluorescence on the Raman spectra, using the airPLS algorithm (Zhangfit) [31]. SNV and autoscale pre-processing were furtherly applied for extracting the spectral information from Raman spectroscopic data. In a similar way, fluorescence spectroscopic data and the visible absorption data were processed with a combination of Savitzky-Golay [44], SNV and autoscale pre-processing techniques. 

To avoid information leakage from the test set, both for the FAMEs and spectroscopic data, the autoscale procedure was performed independently on each feature of the samples in the train set. The statistics were stored in order to be used on the test data. 

## 4. Conclusions

In this work, a thorough study has been performed for the varietal identification of Greek EVOOs. Different analytical techniques have been implemented and compared, from more conventional ones such as gas chromatographic FAMEs’ determination, to more sophisticated ones, such as spectrometric (i.e., HRMS) and spectroscopic techniques (i.e., visible absorption, Raman and fluorescence spectroscopy). The analytical results were combined with Machine Learning algorithms for the classification of the samples to Koroneiki, Kolovi and Adramytiani varieties. For each technique, the predictive model with the best hyperparameter set was chosen. The models’ predictive power was evaluated on test data. The results indicated that the FAMES and HRMS analysis achieve the highest predictive ability. In parallel, the spectroscopic techniques exhibited high performance with a small number of misclassifications. The models were furtherly tested using commercially available market EVOOs for the verification of their variety. The results demonstrated that both targeted and untargeted techniques correctly predicted the variety of all the commercially available market EVOOs, except from the visible absorption spectroscopy, where only 3 out of the 30 market EVOOs were misclassified. 

Based on these encouraging results, HRMS metabolomics as well as optical spectroscopic techniques combined with machine learning methods provide an alternative approach for the identification of olive oil variety, compared to the official methods already established (e.g., FAMEs). Through untargeted metabolomics a thorough investigation on EVOOs chemical profile may be accomplished, while that identification of compounds of interest is also enabled, exploiting HRMS full capabilities. Regarding optical techniques, they have prevailed in the field as they are facile, rapid and cost-effective compared to other analytical techniques. They also require no sample pre-treatment, while their predictive performance is comparable to the modern analytical procedures.

Finally, to the best of the authors’ knowledge, this is the first time where different techniques from the fields of standard analysis, spectrometry and spectroscopy are applied to the same EVOO samples, and the results are compared and evaluated by machine learning techniques. The findings of this work are valuable for providing a novel methodology for EVOO authentication that might help in the field of production, packaging and trading of EVOO. 

## Figures and Tables

**Table 1:** Confusion Matrix of FAMEs test samples. SVM was performed and achieved 100% accuracy, mean sensitivity and mean specificity.

Predicted Label				
**True Label**		**Adramytiani**	**Kolovi**	**Koroneiki**
	**Adramytiani**	3	0	0
	**Kolovi**	0	12	0
	**Koroneiki**	0	0	37

**Table 2:** Confusion matrix of HRMS-untargeted metabolomics test samples. Feature Selection using Random Forest combined with Logistic Regression as a classification method, where all samples were correctly classified, leading to 100% accuracy, sensitivity and specificity.

Predicted Label				
**True Label**		**Adramytiani**	**Kolovi**	**Koroneiki**
	**Adramytiani**	3	0	0
	**Kolovi**	0	12	0
	**Koroneiki**	0	0	37

**Table 3:** Confusion matrix of visible absorption spectroscopic test samples. Feature Selection using Random Forest combined with SVM as a classification method was performed. Five out of the total 52 test samples were misclassified leading to 90% accuracy, 95% mean sensitivity and 96% mean specificity.

Predicted Label				
**True Label**		**Adramytiani**	**Kolovi**	**Koroneiki**
	**Adramytiani**	3	0	0
	**Kolovi**	0	12	0
	**Koroneiki**	1	4	32

**Table 4:** Raman band assignments of the EVOO (spectral region 1000–1700 cm^−1^).

Raman Bands (cm^−1^)	Assignments
1072	C–C stretching of (CH_2_)_n_ group
1265	=C–H stretching of cis (R–HC=CH–R)
1300	C–H bending (twist) of CH_2_ group
1440	C–H bending (scissoring) of CH_2_
1655	C=C stretching of (RHC=CHR)

**Table 5:** Confusion matrix of Raman spectroscopic test samples. Feature Selection using Random Forest combined with SVM as a classification method was performed. Three out of 52 test samples were misclassified leading to 94% accuracy and 85% mean sensitivity and 97% mean specificity.

Predicted Label				
**True Label**		**Adramytiani**	**Kolovi**	**Koroneiki**
	**Adramytiani**	2	1	0
	**Kolovi**	1	11	0
	**Koroneiki**	0	1	36

**Table 6:** Confusion matrix of fluorescence spectroscopic test samples. Feature Selection using Random Forest combined with KNN as a classification method was performed. One out of the total 52 samples was misclassified, leading to 98% accuracy, 89% mean sensitivity and 99% mean specificity.

Predicted Label				
**True Label**		**Adramytiani**	**Kolovi**	**Koroneiki**
	**Adramytiani**	2	1	0
	**Kolovi**	0	12	0
	**Koroneiki**	0	0	37

**Table 7:** Accuracy and balanced accuracy values for the techniques used in this study.

	FAMEs	HRMS	Absorption Spectroscopy (400–700 nm)	Raman Spectroscopy	Fluorescence Spectroscopy
**Accuracy (%)**	100	100	90	94	98
**Mean Sensitivity (%)**	100	100	95	85	89
**Mean Specificity (%)**	100	100	96	97	99

**Table 8:** Number of correctly classified market samples. The total number of market samples was 30.

	FAMEs	HRMS	Absorption Spectroscopy (400–700 nm)	Raman Spectroscopy	Fluorescence Spectroscopy
**#correctly identified**	**30**/30	**30**/30	**27**/30	**30**/30	**30**/30

## Data Availability

Not applicable.

## Supplementary Materials

The following are available online. Table S1: Fatty acids detected in extra virgin olive oil along with their categorization in MUFAs and PUFAs based on their saturation, Table S2: Single factor ANOVA (alpha = 0.05) of FAMEs for EVOOs of different varieties. *p*-value below 0.05 denotes that the mean difference between the groups is statistically significant, Table S3: Single factor ANOVA (alpha = 0.05) of FAMEs between the two groups of Koroneiki variety. In the case of Koroneiki (2018–2020) and Koroneiki (market) EVOOs, the *p*-value above 0.05 denotes that the mean difference between the groups is not statistically significant, both in MUFAs (A) and PUFAs (B), Table S4: Statistical parameters of fatty acids (MUFAs, PUFAs, linoleic acid and alpha-linolenic acid) in EVOOs of different variety, as well as in samples from the market, Table S5: Compounds detected in EVOOs, with the target database, in negative acquisition mode ([M–H]^−^), Table S6: Single factor ANOVA (alpha = 0.05) of bioactive content for EVOOs of different varieties. *p*-value below 0.05 denotes that the mean difference between the groups is statistically significant, Table S7: Statistical parameters of bioactive content in EVOOs of different variety, as well as in samples from the market, Table S8: Tentative identification of characteristic markers in EVOOs variety classification, Table S9: Sensitivity and Specificity results for each variety, Figure S1: QC charts of EMRT known compounds for (A) Hydroxytyrosol (153.0557_3.5), (B) Naringenin (271.0612_7.0) and (C) Oleacein (319.1187_5.6), Table S10: Hyperparameters of feature selection and classification methods.
