# Supplementary material for: Comparative Evaluation of Different Targeted and Untargeted Analytical Approaches to Assess Greek Extra Virgin Olive Oil Quality and Authentication
Source: Molecules. 2022 Feb 16;27(4):1350. doi: 10.3390/molecules27041350 (PMC8874659; doi:10.3390/molecules27041350)
Supplement: Supplementary file 1 [file molecules-27-01350-s001.zip › molecules-1443765-supplementary.pdf]

SUPPORTING INFORMATION

Comparative Evaluation of Different Targeted and Untargeted Analytical Approaches to Assess Greek Extra Virgin Olive Oil Quality and Authentication

Sofia Drakopoulou [1,†], Emmanouil Orfanakis [2,3,†], Ioulia Karagiannaki [2], Fragiskos Gaitis [4], Stavroula Skoulika [4], Andreas Papaioannou [4], George Boukouvalas [4], George Petropoulos [4], Vassilios Katsoudas [4], Renate Kontzedaki [2], Aggelos Philippidis [2], Aikaterini Zoumi [2], Marilena Dasenaki [1,5], Nikolaos Thomaidis [1,*,‡] and Michalis Velegrakis [2,*,‡]

[1] Laboratory of Analytical Chemistry, Department of Chemistry, National and Kapodistrian University of Athens, Panepistimiopolis Zografou, 15771 Athens, Greece; sofiadrakop@chem.uoa.gr (S.D.); mdasenaki@chem.uoa.gr (M.D.)
[2] Institute of Electronic Structure and Laser, Foundation for Research and Technology-Hellas (IESL-FORTH), 70013 Heraklion, Crete, Greece; morfanakis@iesl.forth.gr (E.O.); ikaragian@iesl.forth.gr (I.K.); renatekontzedaki@iesl.forth.gr (R.K.); filagg@iesl.forth.gr (A.P.); azoumi@iesl.forth.gr (A.Z.)
[3] Department of Materials Science and Technology, University of Crete, 70013 Heraklion, Crete, Greece
[4] Food Analytical and Research Laboratories of Athens, Directorate of Laboratories, Hellenic Food Authority (EFET) 143 42, 31 Anagenniseos str, Nea Philadelfeia, 11526 Athens, Greece; fgaitis@efet.gr (F.G.); sskoulika@efet.gr (S.S.); apapaioannou@efet.gr (A.P.); gboukouvalas@efet.gr (G.B.); gpetropoulos@efet.gr (G.P.); vkatsoudas@efet.gr (V.K.)
[5] Laboratory of Food Chemistry, Department of Chemistry, National and Kapodistrian University of Athens, Panepistimiopolis Zografou, 15771 Athens, Greece
† These authors contributed equally as co-first authors.
‡ These authors contributed equally as co-last authors.
* Correspondence: ntho@chem.uoa.gr (N.S.T.); vele@iesl.forth.gr (M.V.); Tel: +30-210-7274317 (N.T.), +30-281-0391122 (M.V.)

SI-2. Results and Discussion

SI-2.1. Fatty acids methyl esters (FAMEs)

Table S1. Fatty acids detected in extra virgin olive oil along with their categorization in MUFAs and PUFAs based on their saturation.

	Fatty Acid	Abbreviation	Categorization (by the Extent of Saturation)
1	Palmitic acid	C16:1	MUFA
2	Decaheptanoic acid	C17:1	MUFA
3	Oleic acid	C18:1	MUFA
4	Eicosenoic acid	C20:1	MUFA
5	Erucic acid	C22:1	MUFA
6	trans oleic acid	C18:1	MUFA
7	Linoleic acid	C18:2	PUFA
8	alpha-linolenic acid	C18:3	PUFA
9	Total trans linoleic + trans linolenic isomers	C18:2 + C18:3	PUFA
10	Lauric acid	C12:0	SFA
11	Myristic acid	C14:0	SFA

12	Palmitic acid	C16:0	SFA
13	Heptadecanoic acid	C17:0	SFA
14	Stearic acid	C18:0	SFA
15	Arachidonic acid	C20:0	SFA
16	Behenic acid	C22:0	SFA
17	Lignoceric acid	C24:0	SFA

MUFA: monounsaturated fatty acid. PUFA: polyunsaturated fatty acid. SFA: saturated fatty acid.

Table S2. Single factor ANOVA (alpha=0.05) of FAMEs for EVOOs of different varieties. P-value below 0.05 denotes that the mean difference between the groups is statistically significant, both in MUFAs (A) and PUFAs (B).

(A)

Anova: Single Factor

SUMMARY

Groups	Count	Sum	Mean	Variance
Adramytiani	8	559	69.9	1.00
Kolovi	41	3085	75.2	8.89
Koroneiki (2018-2020)	124	9496	76.6	4.49
Koroneiki (market)	30	2292	76.4	1.66

ANOVA

Source of Variation	SS	df	MS	F	P-value	F crit
Between Groups	365	3	122	25.1	7.78E-14	2.65
Within Groups	963	199	4.84			
Total	1328	202				

(B)

Anova: Single Factor

SUMMARY

Groups	Count	Sum	Mean	Variance
Adramytiani	8	109	13.7	0.43
Kolovi	41	464	11.3	3.93
Koroneiki (2018-2020)	124	930	7.50	2.92
Koroneiki (market)	30	238	7.92	0.83

ANOVA

Source of Variation	SS	df	MS	F	P-value	F crit
Between Groups	671	3	224	82.0	1.46E-34	2.65
Within Groups	543	199	2.73			
Total	1215	202				

Table S3. Single factor ANOVA (alpha=0.05) of FAMEs between the two groups of Koroneiki variety. In the case of Koroneiki (2018-2020) and Koroneiki (market) EVOOs, the P-value above 0.05 denotes that the mean difference between the groups is not statistically significant, both in MUFAs (A) and PUFAs (B).

(A)

Anova: Single Factor

SUMMARY

Groups	Count	Sum	Mean	Variance
Koroneiki (2018-2020)	124	9496	76.6	4.49
Koroneiki (market)	30	2292	76.4	1.66

ANOVA

Source of Variation	SS	df	MS	F	P-value	F crit
Between Groups	0.93	1	0.93	0.23	0.63	3.90
Within Groups	600	152	3.95			
Total	601	153				

(B)

Anova: Single Factor

SUMMARY

Groups	Count	Sum	Mean	Variance
Koroneiki (2018-2020)	124	930	7.50	2.92
Koroneiki (market)	30	238	7.92	0.83

ANOVA

Source of Variation	SS	df	MS	F	P-value	F crit
Between Groups	4.25	1	4.25	1.69	0.20	3.90
Within Groups	383	152	2.52			
Total	387	153				

Table S4. Statistical parameters of fatty acids (MUFAs, PUFAs, linoleic acid and alpha-linolenic acid) in EVOOs of different variety, as well as in samples from the market.

	**MUFAs (g/100g)**			
	Adramytiani (n=8)	Kolovi (n=41)	Koroneiki (2018-2020) (n=124)	Koroneiki (market) (n=30)
Median	69.8	75.6	76.8	76.4
Mean	69.9	75.2	76.6	76.4
Standard Deviation	1.0	3.0	2.1	1.3
Range	68.8-71.5	64.8-79.1	64.8-79.7	74.2-79.2
	**PUFAs (g/100g)**			
Median	13.7	11.2	7.23	7.89
Mean	13.7	11.3	7.50	7.92
Standard Deviation	0.7	2.0	1.7	0.9
Range	12.4-14.8	6.19-17.3	5.03-17.3	6.68-9.75
	**Linoleic acid (g/100g)**			
Median	12.9	10.3	6.46	7.14
Mean	12.8	10.4	6.72	7.17
Standard Deviation	0.6	1.9	1.7	0.9
Range	11.7-13.8	5.55-16.3	4.41-16.3	5.96-9.14
	**alpha-Linolenic acid (g/100g)**			

Median	0.82	0.82	0.75	0.72
Mean	0.81	0.85	0.75	0.72
Standard Deviation	0.1	0.1	0.1	0.1
Range	0.66-0.95	0.59-1.23	0.55-0.98	0.59-0.90

SI-2.2 HRMS-metabolomics

Table S5. Compounds detected in EVOOs, with the target database, in negative acquisition mode ($[M-H]^-$).

Compound	Molecular Formula	m/z (Precursor Ion)	t_R (min)	Fragments
10-Hydroxy decarboxymethyl oleuropein aglycone*	$C_{17}H_{20}O_7$	335.1125	4.28	199.0613; 151.0401; 121.0292
10-Hydroxy-10-Methyl oleuropein aglycone*	$C_{20}H_{24}O_9$	407.1348	6.71	195.0665; 111.0087
10-Hydroxyoleuropein aglycone*	$C_{19}H_{22}O_9$	393.1191	4.82	181.0502; 137.0244
1-Acetoxypinoresinol	$C_{22}H_{24}O_8$	415.1398	6.42	151.0402; 343.1188; 280.0951
Apigenin	$C_{15}H_{10}O_5$	269.0455	7.90	117.0346; 151.0037; 149.0244
Elenolic acid	$C_{11}H_{14}O_6$	241.0718	4.51	95.0496; 127.0401; 171.0300
Eriodictyol	$C_{15}H_{12}O_6$	287.0561	6.40	151.0037; 135.0452
Hydroxylated form of elenolic acid	$C_{11}H_{14}O_7$	257.0667	1.36	137.0603
Hydroxytyrosol*	$C_8H_{10}O_3$	153.0557	3.47	123.0451
Hydroxytyrosol acetate*	$C_{10}H_{12}O_4$	195.0663	6.71	149.0608
Ligstroside aglycone*	$C_{19}H_{22}O_7$	361.1293	8.50	101.0244; 127.0401
Luteolin	$C_{15}H_{10}O_6$	285.0405	7.10	133.0295; 151.0037
Methyl oleuropein aglycone*	$C_{20}H_{24}O_8$	391.1398	7.51	67.0192
Naringenin	$C_{15}H_{12}O_5$	271.0612	7.00	119.0502; 151.0037
Oleacein*	$C_{17}H_{20}O_6$	319.1187	5.60	139.0765
Oleocanthal*	$C_{17}H_{20}O_5$	303.1238	6.55	69.0346; 137.0608
Oleocanthalic acid*	$C_{17}H_{20}O_6$	319.1187	5.00	111.0088; 199.0612
Oleokoronal*	$C_{19}H_{22}O_7$	361.1293	6.80	259.0975; 291.0875
Oleomissional*	$C_{19}H_{22}O_8$	377.1242	5.95	95.0502; 139.0037
Oleuropein aglycone*	$C_{19}H_{22}O_8$	377.1242	7.40	95.0502; 111.0088; 139.0037
p-coumaric acid	$C_9H_8O_3$	163.0400	2.77	119.0502
Pinoresinol	$C_{20}H_{22}O_6$	357.1344	6.45	151.0401
Syringaresinol	$C_{22}H_{26}O_8$	417.1555	6.18	181.0506; 127.0408
Tyrosol*	$C_8H_{10}O_2$	137.0608	4.11	119.0502
Vanillin	$C_8H_8O_3$	151.0401	4.60	108.0217; 136.0166

* Hydroxytyrosol derivatives (co-estimated in bioactive content as established by EU 432/2012 legislation [1].

Table S6. Single factor ANOVA (alpha=0.05) of bioactive content for EVOOs of different varieties. P-value below 0.05 denotes that the mean difference between the groups is statistically significant.

**Anova: Single Factor**

SUMMARY

Groups	Count	Sum	Mean	Variance
Adramytiani	8	283	35.4	1103
Kolovi	41	11193	273	25225
Koroneiki (2018-2020)	124	44157	356	116404
Koroneiki (market)	30	4951	165	10659

ANOVA

Source of Variation	SS	df	MS	F	P-value	F crit
Between Groups	1526477	3	508826	6.47	3.35E-04	2.65
Within Groups	15643594	199	78611			
Total	17170071	202				

Table S7. Statistical parameters of bioactive content in EVOOs of different variety, as well as in samples from the market.

	Bioactive content (mg/kg)			
	Adramytiani (n=8)	Kolovi (n=41)	Koroneiki (2018-2020) (n=124)	Koroneiki (market) (n=30)
Median	18.7	238	255	154
Mean	35.4	273	356	165
Standard Deviation	33	159	341	103
Range	8.24-96.8	31.3-639	36.3-2168	26.7-445

Table S8. Tentative identification of characteristic markers in EVOOs variety classification.

**EMRT features m/z meas._R_t (min)**	**Ion**	**Mass Error (mDa)**	**Fragments (m/z)**	**Probable Elemental Composition**	**Tentative Identification**	**Marker**
163.0402_2.40	$[M-H]^-$	0.7	119.0502	$C_9H_8O_3$	p-coumaric acid [1]	Adramytiani
195.0662_7.04	$[M-H]^-$	0.5	149.0608	$C_{10}H_{12}O_4$	Hydroxytyrosol acetate [1]	Kolovi
229.1081_4.38	$[M-H]^-$	0.5	169.0846<br>155.0690	$C_{11}H_{18}O_5$	DEDA acetal	Koroneiki
253.2171_13.15	$[M-H]^-$	1.3	-	$C_{16}H_{30}O_2$	Palmitoleic acid [2]	Adramytiani
287.0557_6.26	$[M-H]^-$	-0.1	135.0452<br>151.0037	$C_{15}H_{12}O_6$	Eriodictyol [1]	Kolovi
311.2223_9.95	$[M-H]^-$	0.8	-	$C_{18}H_{32}O_4$	Octadecenedioate [2]	Adramytiani
361.1289_9.15	$[M-H]^-$	1.4	101.0244<br>127.0401	$C_{19}H_{22}O_7$	Ligstroside aglycone isomer [1]	Koroneiki
375.1084_7.04	$[M-H]^-$	0.6	195.0673<br>153.0559<br>127.0419	$C_{19}H_{20}O_8$	Dehydro oleuropein aglycone	Kolovi
377.1240_7.38	$[M-H]^-$	1.3	111.0088<br>139.0037	$C_{19}H_{22}O_8$	Oleuropein aglycone isomer [1]	Koroneiki
455.3527_13.85	$[M-H]^-$	1.7	-	$C_{30}H_{48}O_3$	Oleanolic acid [2]	Koroneiki
471.3475_12.83	$[M-H]^-$	0.4	-	$C_{30}H_{48}O_4$	Maslinic acid [1]	Koroneiki

[1] Annotated according to target database.
[2] Annotated according to suspect database [2].

SI-2.3 Optical Spectroscopic Methods

Table S9. Sensitivity and Specificity results for each variety.

		Adramytiani (n=8)	Kolovi (n=41)	Koroneiki (2018-2020) (n=124)	**Mean**
FAMEs	Sensitivity	1	1	1	1
	Specificity	1	1	1	1
HRMS	Sensitivity	1	1	1	1
	Specificity	1	1	1	1
Absorption	Sensitivity	1	1	0.864	0.954
	Specificity	0.979	0.9	1	0.959
Fluorescence	Sensitivity	0.666	1	1	0.888
	Specificity	1	0.975	1	0.991
Raman	Sensitivity	0.666	0.916	0.972	0.851
	Specificity	0.979	0.95	1	0.976

SI-3. Materials and Methods

SI-3.2. EVOOs Samples and Sample Preparation Protocols

(A)

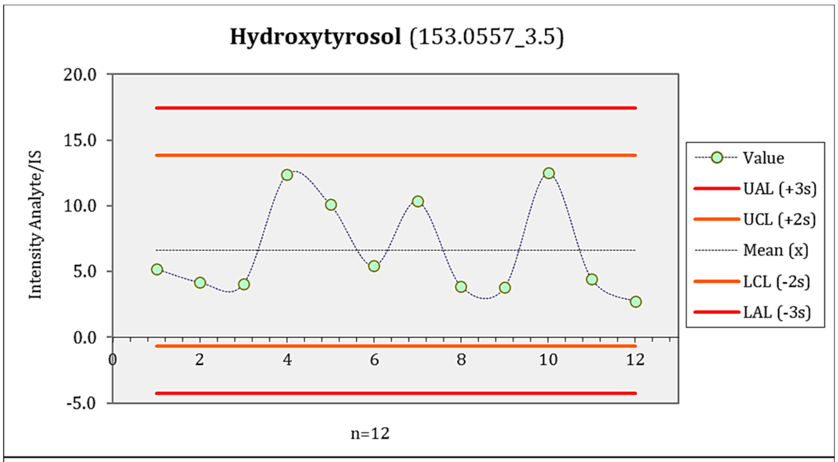

(B)

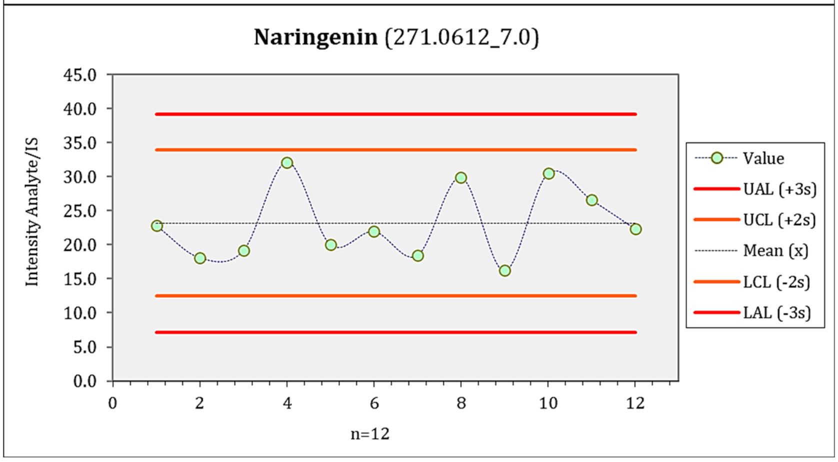

(C)

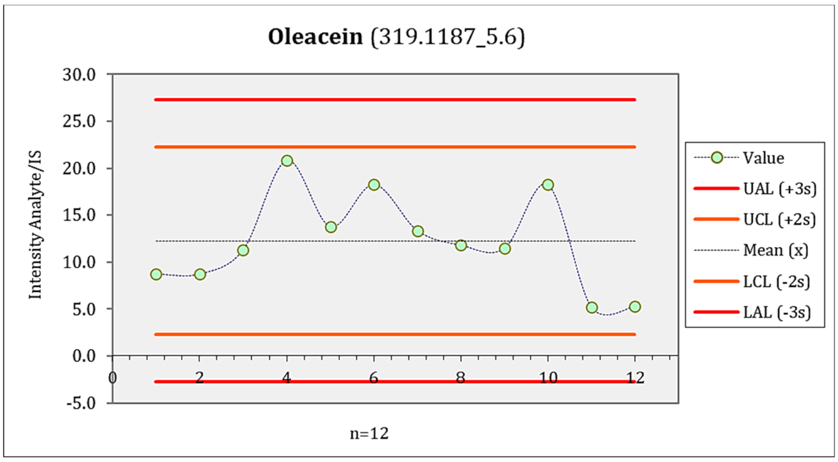

Figure S1. QC charts of EMRT known compounds for (**A**) Hydroxytyrosol (153.0557_3.5), (**B**) Naringenin (271.0612_7.0) and (**C**) Oleacein (319.1187_5.6).

SI-3.5. Machine Learning Analysis

SI-3.5.1. Methods

Table S10. Hyperparameters of feature selection and classification methods.

**Feature Selection**	
**Random Forest (RF)**	
**Number of trees**	100
**Minimum split size**	2, 3, 5, 9, 10
**Minimum leaf size**	2, 3, 5, 9, 10
**Classification**	
**Support Vector Machines (SVM)**	
**Kernel**	Linear, Radial Basis Function (RBF), Polynomial, Sigmoid
**C**	0.00001, 0.0001, 0.001, 0.01, 0.5, 1, 1.5, 2, 3
**Random Forest (RF)**	
**Number of trees**	100
**Minimum split size**	2, 3, 5, 9, 10
**Minimum leaf size**	2,3,5,9,10
**K Nearest Neighbors (KNN)**	
**k**	3,5,7,10,15, 20
**Logistic Regression (LR)**	
**C**	0.00001, 0.0001, 0.001, 0.01, 0.5, 1, 1.5, 2, 3
**Penalty**	L1, L2

References

1. European Commission Commission Regulation (EU) No 1018/2013 of 23 October 2013 amending Regulation (EU) No 432/2012 establishing a list of permitted health claims made on foods other than those referring to the reduction of disease risk and to children's development and heal. *Off. J. Eur. Union L 282* **2013**, *56*, 43–45, doi:http://eur-lex.europa.eu/pri/en/oj/dat/2003/l_285/l_28520031101en00330037.pdf.
2. Kalogiouri, N.P.; Aalizadeh, R.; Thomaidis, N.S. Application of an advanced and wide scope non-target screening workflow with LC-ESI-QTOF-MS and chemometrics for the classification of the Greek olive oil varieties. *Food Chem.* **2018**, *256*, 53–61, doi:10.1016/j.foodchem.2018.02.101.
